# Simulation Evidence of Hexagonal‐to‐Tetragonal ZnSe Structure Transition: A Monolayer Material with a Wide‐Range Tunable Direct Bandgap

**DOI:** 10.1002/advs.201500290

**Published:** 2015-10-28

**Authors:** Lei Li, Pengfei Li, Ning Lu, Jun Dai, Xiao Cheng Zeng

**Affiliations:** ^1^ Department of ChemistryUniversity of Nebraska‐LincolnLincolnNE68588USA; ^2^ Hefei National Laboratory for Physical Sciences at Microscale and Collaborative Innovation Center of Chemistry for Energy MaterialsUniversity of Science and Technology of ChinaHefeiAnhui230026P.R. China

**Keywords:** light emitting and absorption, strain engineering, tetragonal ZnSe monolayer, wide‐range tunable direct bandgap

## Abstract

2D material with tunable direct bandgap in the intermediate region (i.e., ≈2–3 eV) is key to the achievement of high efficiency in visible‐light optical devices. Herein, a simulation evidence of structure transition of monolayer ZnSe from the experimental pseudohexagonal structure to the tetragonal structure (t‐ZnSe) under lateral pressure is shown, suggesting a possible fabrication route to achieve the t‐ZnSe monolayer. The as‐produced t‐ZnSe monolayer exhibits highly tunable bandgap under the biaxial strains, allowing strain engineering of t‐ZnSe's bandgap over a wide range of 2–3 eV. Importantly, even under the biaxial strain up to 7%, the t‐ZnSe monolayer still keeps its direct‐gap property in the desirable range of 2.40–3.17 eV (corresponding to wavelength of green light to ultraviolet). The wide‐range tunability of direct bandgap appears to be a unique property of the t‐ZnSe monolayer, suggesting its potential application as a light‐emitting 2D material in red–green–blue light emission diodes or as complementary light‐absorption material in the blue–yellow region for multijunction solar cells. The straddling of the band edge of the t‐ZnSe monolayer over the redox potential of water splitting reaction also points to its plausible application for visible‐light‐driven water splitting.

## 1 Introduction

2D materials consist of few atomic layers are not only highly flexible, light in weight, and susceptible to large‐scale manufacture but also can possess novel electronic and optical properties that are unattainable in bulk counterparts.^1^, ^2^, ^3^, ^4^ These advantages open the possibility for using 2D materials in future generations of electronic and optical devices. Examples of 2D materials with direct bandgaps include graphene analogs such as inorganic transition‐metal dichalcogenides (TMDCs), transition‐metal trichalcogenides, and phosphorene which have been demonstrated or predicted as channel materials in field‐effect transistors (FETs) or as light‐detector or light–emitting optoelectronic materials.^5^, ^6^, ^7^, ^8^, ^9^, ^10^, ^11^, ^12^, ^13^, ^14^, ^15^, ^16^ Some limitations, however, are still present for high‐performance application of 2D materials in devices, such as carrier mobility reduction, high contact resistance, and low light absorption and emission efficiency.^10^, ^17^

For optoelectronic application of 2D materials, in particular, continuously tunable and direct bandgap over a wide range is highly desirable to achieve wide‐spectrum optical absorption and high photoluminescence efficiency.^2^, ^18^, ^19^, ^20^ Moreover, because strong intensity of the sunlight is mainly in the yellow–blue region (2.0–2.85 eV), new 2D materials with tunable bandgaps that are in the yellow–blue‐light range will be more advantageous as light‐absorbing materials for optoelectronic application. As an example, the energy range of 2.3–2.5 eV is crucial to achieve high efficiencies (>50%) in multijunction solar cells.^21^ To our knowledge, few binary compounds possess bandgaps in this intermediate range.^21^, ^22^ Also, display or lighting materials generally require integration of red, green, and blue LEDs into a single device as the red–green–blue (RGB) LEDs. However, most 2D materials reported thus far entail a bandgap either less than 2.0 eV (e.g., MoS_2_, TiS_3_, and black phosphorus)^1^, ^4^, ^23^ or greater than 3.0 eV (i.e. BN).^24^ For example, 2D MoS_2_, MoSe_2_, and WSe_2_ exhibit electroluminescence within the red‐light region (<2.0 eV).^9^, ^25^, ^26^, ^27^, ^28^ Few reports have been seen in the literature on 2D materials that can emit green and blue lights. Furthermore, integration of RGB LEDs from different materials can be challenging in conventional nitride‐based LEDs due to different fabrication conditions and starting materials used for the different materials. Here, we show theoretical evidence that t‐ZnSe is a novel 2D material that possesses a continuously tunable bandgap covering the blue‐light region to the red‐light region.

Recently, Sun et al. reported successful synthesis of freestanding monolayers of nonlayered ZnSe with four‐atomic thickness.^29^ The as‐synthesized ZnSe monolayers exhibit pseudohexagonal lattice structure. The pseudohexagonal ZnSe (ph‐ZnSe) monolayers give much enhanced photocurrent density, ≈8 and ≈200 times higher than that of ZnSe quantum dots and bulk ZnSe, respectively. Moreover, the ph‐ZnSe monolayer possesses a direct bandgap of ≈3.50 eV such that it could be an effective photocatalyst for water splitting by absorbing ultraviolet light. Using density functional theory (DFT) methods, Tong et al. and Zhou et al. showed that a novel tetragonal ZnSe (t‐ZnSe) monolayer is more stable than the ph‐ZnSe monolayer, and is expected to exhibit comparable photocatalytic activity in ultraviolet region.^30^, ^31^ However, direct simulation evidence on the formation of the tetragonal structure is still lacking. Moreover, the bandgap tunability of both types of ZnSe monolayer is still unresolved. It will be desirable that the direct bandgap can be reduced via strain engineering so that absorption of visible light can be achieved. In this study, by using the Born–Oppenheimer molecular dynamics (BOMD) simulation,^32^, ^33^ we show a direct simulation evidence of structure transition from the pseudohexagonal to tetragonal structure, suggesting a possible synthesis route for producing the t‐ZnSe monolayer from the metastable ph‐ZnSe monolayer. Our global minimum search also shows that the t‐ZnSe monolayer is the lowest‐energy 2D structure. Importantly, it can exhibit a ‘continuously tunable' bandgap, ranging from 3.17 to 1.97 eV, while maintaining the direct‐gap semiconducting properties under the biaxial strain up to 7%. In contrast, the ph‐ZnSe monolayer tends to retain its bandgap value (≈3.40 eV) even under tensile strain as high as 10%.

## 2 Results and Discussion

### 2.1 Global Minimum Search and Geometric Properties of ZnSe Monolayers

**Figure 1:**
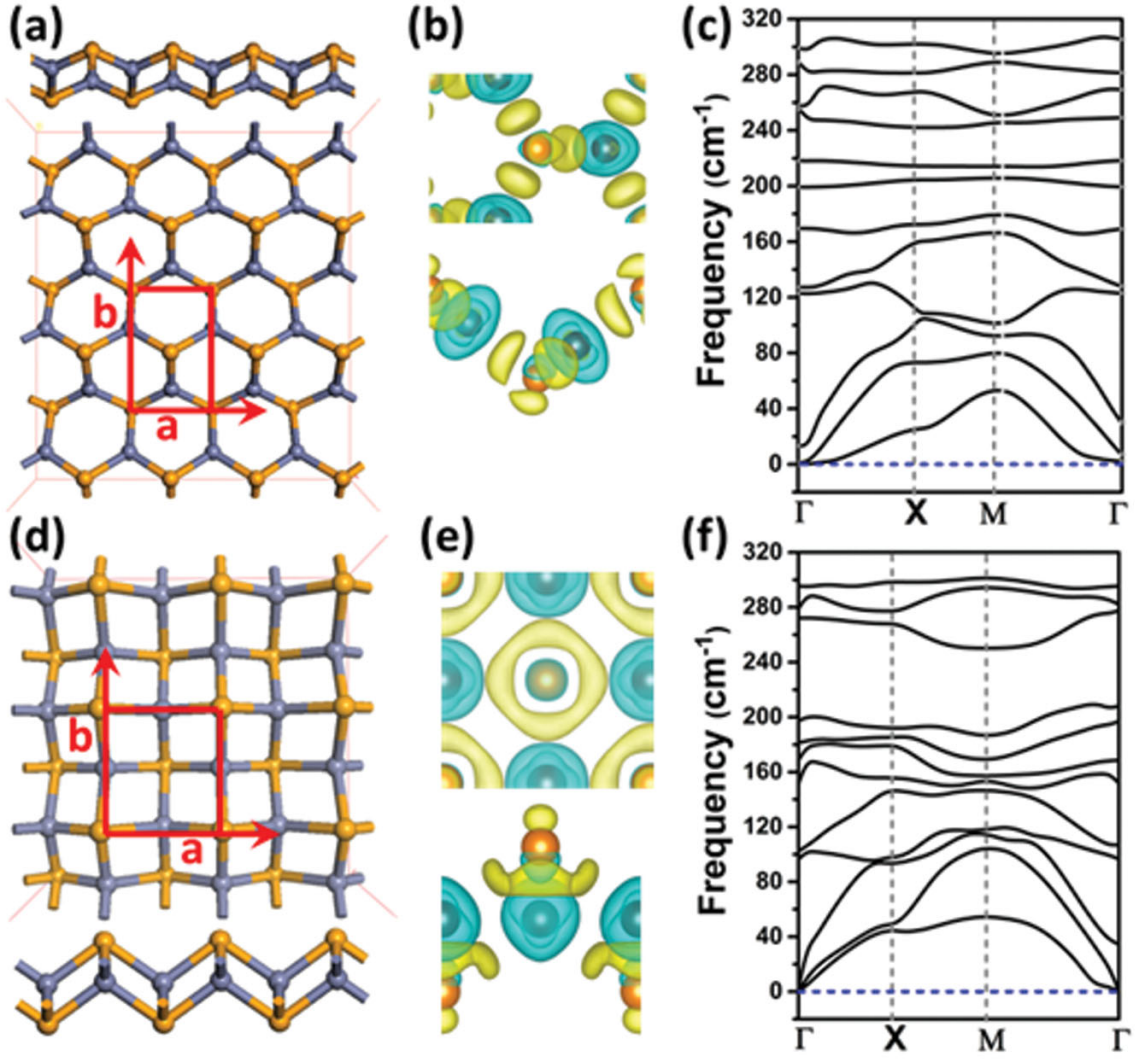
a) Optimized structure, b) computed deformation charge density, and c) phonon spectrum of the ph‐ZnSe monolayer. The corresponding computation results for t‐ZnSe are displayed in panels (d–f), respectively. In panels (a) and (d), both top and side views of the optimized structure are given. The gray and orange spheres represent Zn and Se atoms, respectively. The unit cell of the ZnSe monolayer is highlighted in red. The deformation charge densities are plotted with isosurface value of 0.008 e Bohr^−3^, where the yellow and blue represent the electron‐rich and electron‐deficient regions, respectively.

The global minimum structure search for the lowest‐energy 2D ZnSe monolayer is carried out using the CALYPSO software package.^34^, ^35^ The obtained three low‐energy structures (see **Figure** **1**a,d and Supporting Information Figure S1) are further optimized using the DFT methods. Next, phonon spectrum of the lowest‐energy ZnSe monolayer is computed to confirm dynamic stability, while BOMD simulations are performed to confirm its thermal stability at an elevated temperature.^36^, ^37^

In Figure 1a,d, the two lowest‐energy structures of ZnSe mono­layers are displayed. The pseudohexagonal structure (Figure 1a) with four‐atomic layers is the same as observed in the experiment.^29^ In the optimized ph‐ZnSe monolayer, the nearest Se–Se and Zn–Se distance is 3.965 and 2.394 Ǻ, respectively, very close to the measured distance (4.012 and 2.457 Å).^29^ Each Zn (Se) atom is surrounded by three Se (Zn) atoms, and the areal density of ph‐ZnSe monolayer is 0.17 atoms Å^−2^. The computed deformation charge density, which reflects the polarity of Zn–Se bonds, indicates that the electron‐rich region is mainly near Se atoms. Computed phonon spectrum of the ph‐ZnSe monolayer (Figure 1c) exhibits no negative frequency, confirming dynamic stability of the ph‐ZnSe monolayer. More interestingly, a more stable ZnSe monolayer with tetragonal structure is revealed (Figure 1d), which is 0.054 eV per unit cell lower in energy (in HSE06 calculation; see Table S1, Supporting Information) than the ph‐ZnSe monolayer. Contrary to the ph‐ZnSe monolayer, the t‐ZnSe monolayer possesses 3‐atomic layers with Zn atomic layer being sandwiched between two Se atomic layers, the same as the structure reported previously.^30^ Here, each Zn atom is bonded with four Se atoms, and the areal density of t‐ZnSe monolayer is 0.24 atoms Å^−2^, much higher than that of the ph‐ZnSe mono­layer. The apparent charge transfer between Zn and Se atoms, as shown in deformation charge difference, suggests formation of the polar Zn–Se bonds. The average Zn–Se bond length is ≈2.536 Å, about 0.135 Å longer than that in the ph‐ZnSe mono­layer. Due to the formation of an extra Zn–Se bond in the unit cell, the t‐ZnSe monolayer is notably lower in energy than the ph‐ZnSe monolayer on the basis of both PW91 and HSE06 computations (Table S1, Supporting Information). Again, no negative frequency is seen in the phonon spectrum (Figure 1f), suggesting dynamic stability of the t‐ZnSe monolayer. The t‐ZnSe monolayer is also thermally stable based on the BOMD simulation in the constant‐temperature and constant‐volume (*NVT*) ensemble. As shown in Movie S1 (Supporting Information), the t‐ZnSe monolayer can maintain its structure integrity at temperature 500 K during the 30 ps BOMD simulation. Also, another independent 28 ps BOMD simulation in the constant‐pressure and constant‐temperature (*NPT*) ensemble (Movie S2, Supporting Information) shows that the t‐ZnSe monolayer is stable under the ambient temperature (300 K) and pressure (1 atm). Overall, we confirm that the t‐ZnSe monolayer with a higher areal density is more stable than the ph‐ZnSe monolayer.

### 2.2 Electronic and Optical Properties of ZnSe Monolayers

**Figure 2:**
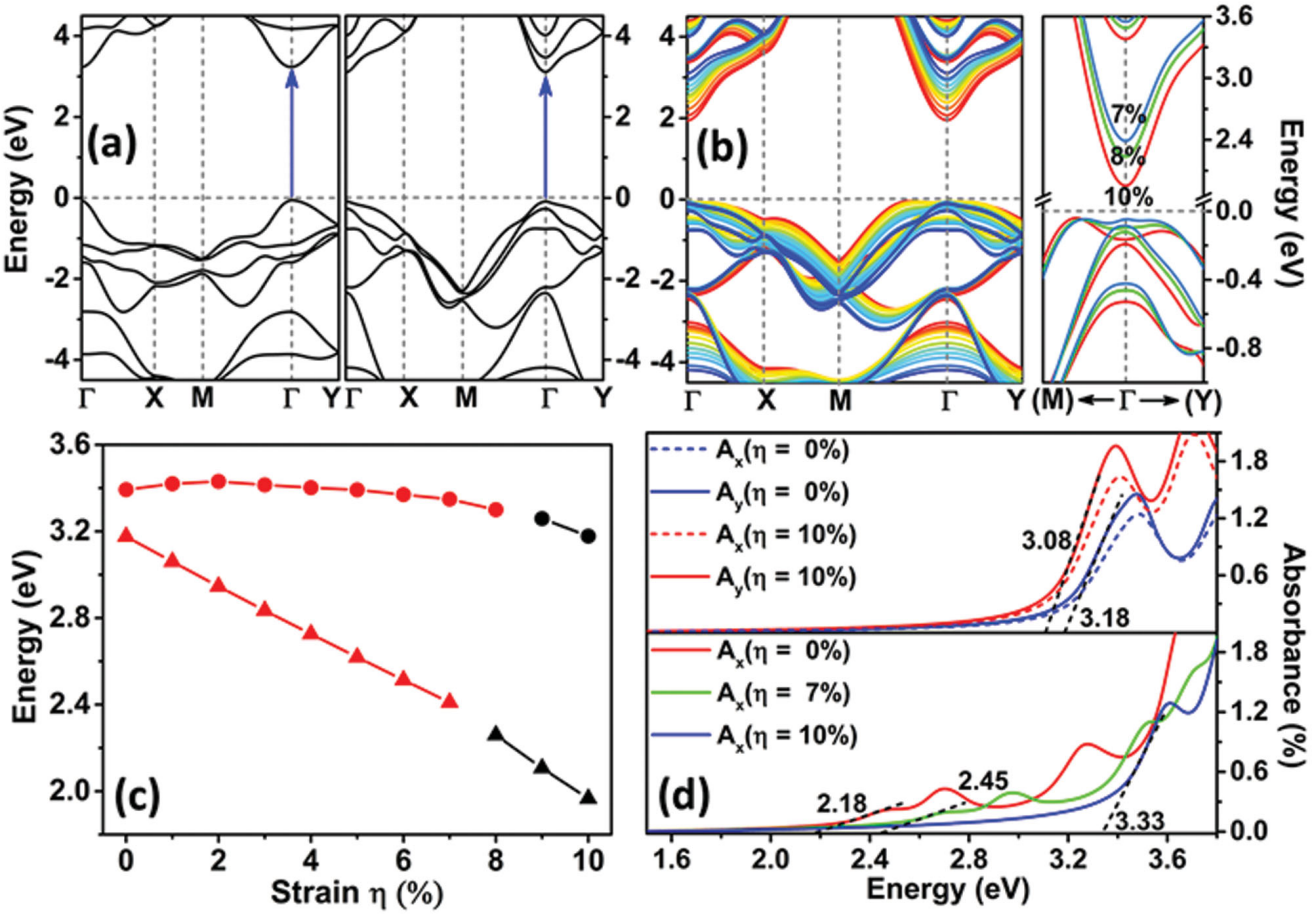
a) Computed band structures of optimized ph‐ZnSe (left panel) and t‐ZnSe (right panel) monolayers. The special points Γ, X, M, and Y refer to (0.0, 0.0, 0.0), (1/2, 0.0, 0.0), (1/2, 1/2, 0.0), and (0.0, 1/2, 0.0), respectively. b) The left panel displays the band structures of the t‐ZnSe monolayer at different biaxial strain from 0% (blue line) to 10% (red line). The right panel presents the zoomed‐in band structures at 7% (light blue line), 8% (green line), and 10% (red line) strain for the t‐ZnSe monolayer near the Γ point. c) Bandgap of the ph‐ZnSe (in circle) and t‐ZnSe (in triangle) monolayers versus the biaxial strain. The direct and indirect bandgaps are distinguished by red and black colors, respectively. d) Strain‐dependent optical absorption spectra of the ph‐ZnSe (upper panel) monolayers for incident light polarized along *a* (*x*) and *b* (*y*) directions and the t‐ZnSe (lower panel) monolayers along the *a* (*x*) direction. The black dashed lines denote approximate linear fitting of the left edge of the first peak whereas the neighboring data represent estimated bandgap.

The electronic and optical properties of ZnSe monolayers are computed based on the HSE06 functional. The HSE06 functional usually predicts more accurate bandgaps and the alignment of the band edges, compared to the Perdew–Burke–Ernzerhof (PBE) functional used previously.^30^, ^31^ As shown in the left panel of **Figure** **2**a, the computed direct bandgap of the ph‐ZnSe monolayer is ≈3.40 eV which is in good agreement with the experimental value (≈3.50 eV),^29^ whereas the bandgap computed at the PBE level is ≈1.36 eV less.^30^, ^31^ The optical absorbance is computed based on the frequency‐dependent dielectric constants at the HSE06 level.^4^, ^38^ For the ph‐ZnSe monolayer the light absorption appears only in the ultraviolet region (upper panel in Figure 2d), consistent with Sun et al.'s result.^29^ A good agreement between the theoretical and experimental results (e.g., bandgap and absorption spectrum) indicates that the HSE06 functional is quite reasonable for the prediction of electronic and optical properties of ZnSe systems.

Like the ph‐ZnSe monolayer, the tetragonal ZnSe monolayer also exhibits semiconducting properties with a direct bandgap of ≈3.17 eV (the right panel of Figure 2a). Also, light absorption appears in the ultraviolet region. However, unlike the ph‐ZnSe monolayer, the bandgap of t‐ZnSe monolayer can be tuned continuously from 3.17 to 2.40 eV by increasing the biaxial strain from 0% to 7% (see Figure 2b,c). Moreover, the bandgap remains to be direct over this strain range (0%–7%), a highly desirable property for achieving high efficiency in light absorption. On the other hand, the ph‐ZnSe monolayer exhibits slight changes in the bandgap up to the strain of 8% (see red circles in Figure 2c). Increasing the strain beyond 8% can induce the direct‐to‐indirect bandgap transition for both types of ZnSe monolayer due to relocation of the valence band maximum (VBM) near the K point (1/3, 1/3, 0.0) for t‐ZnSe (Figure 2b) or near the Σ point (1/5, 1/5, 0.0) for ph‐ZnSe monolayer (Figure S2, Supporting Information). For the t‐ZnSe monolayer, the bandgap can be further reduced to 1.97 eV, whereas for the ph‐ZnSe monolayer the bandgap can still be wider than 3.20 eV. The dramatic difference in bandgap tunability between the t‐ZnSe and ph‐ZnSe monolayers is likely due to the difference in the hybridization of electronic orbitals. In the t‐ZnSe monolayer, the lowest conduction band is mainly contributed by the Zn *s* states (Figure S3, Supporting Information). The strain‐induced elongation of the Zn–Se bonds leads to the downshift of the conduction band, thus resulting in the reduced bandgap. On the other hand, for the ph‐ZnSe monolayer, the Zn *s* and the Se *p* states contribute evenly to the lowest conduction band, indicating strong hybridization of the two states (Figure S4, Supporting Information). Such strong hybridization hinders downshift of the conduction band, and as a result, the bandgap of the ph‐ZnSe monolayer exhibits much weaker tunability. In summary, the bandgap of t‐ZnSe monolayer can be tuned from 3.17 to 1.97 eV due to the weak hybridization between Zn *s* and Se *p* states, whereas the bandgap of ph‐ZnSe monolayer can be reduced by only 0.23 eV via increasing the biaxial strain.

The bandgap reduction due to the strain correlates with the redshift of the absorption spectrum, suggesting that the t‐ZnSe monolayer may be also applicable for photoelectric conversion, such as in solar cells, and for the photocatalytic reaction, such as water splitting. Computed optical absorbance for ph‐ZnSe at 0% and 10% strain and for t‐ZnSe at 0%, 7% and 10% strain are shown in Figure 2d. The energy value obtained from linear fitting of the left edge of the first peak gives rise to the optical gap of the monolayer. The light absorption of the ph‐ZnSe shows light polarization dependence due to the asymmetric structure along *a* and *b* direction. The symmetric structure of the t‐ZnSe results in the same absorption spectra regardless of the polarization. For both types of monolayers at 0% strain, only a peak above 3.0 eV is seen in the absorption spectrum, showing that both ph‐ZnSe and t‐ZnSe monolayers can only absorb ultraviolet light. At 10% strain, no obvious redshift is seen for the ph‐ZnSe monolayer due to little changes in electronic states. However, for the t‐ZnSe monolayer, both 7% and 10% strains lead to two new peaks in the visible‐light region. The edge of the first peak extends to 2.40 and 2.18 eV, respectively, corresponding to the green‐ and yellow‐light regions. So the t‐ZnSe monolayer is expected to absorb visible light due to the strain‐induced bandgap reduction. The absorption in the blue‐yellow region would be critical to the efficiency enhancement for photoelectric conversion, such as in the multijunction solar cells. By combining with other 2D materials with a narrow bandgap (i.e., TMDC monolayer), wide wavelength coverage of light absorption from blue to red light can be achieved to improve the overall efficiency of light conversion.

As mentioned previously, the ph‐ZnSe monolayer has been synthesized and demonstrated as a photocatalyst for water splitting reaction.^29^ However, due to the poor tunability of its bandgap by strain, the ph‐ZnSe monolayer can only absorb the ultraviolet light (3%–5% of the sunlight in energy) but not the visible light which carries 42%–43% of the sunlight energy. On the other hand, the direct bandgap of the t‐ZnSe monolayer can be effectively reduced to visible‐light region (e.g., blue–green‐light region) under suitable biaxial strain. Hence, the t‐ZnSe monolayer is expected to be a more efficient photocatalyst compared to the ph‐ZnSe monolayer.

**Figure 3:**
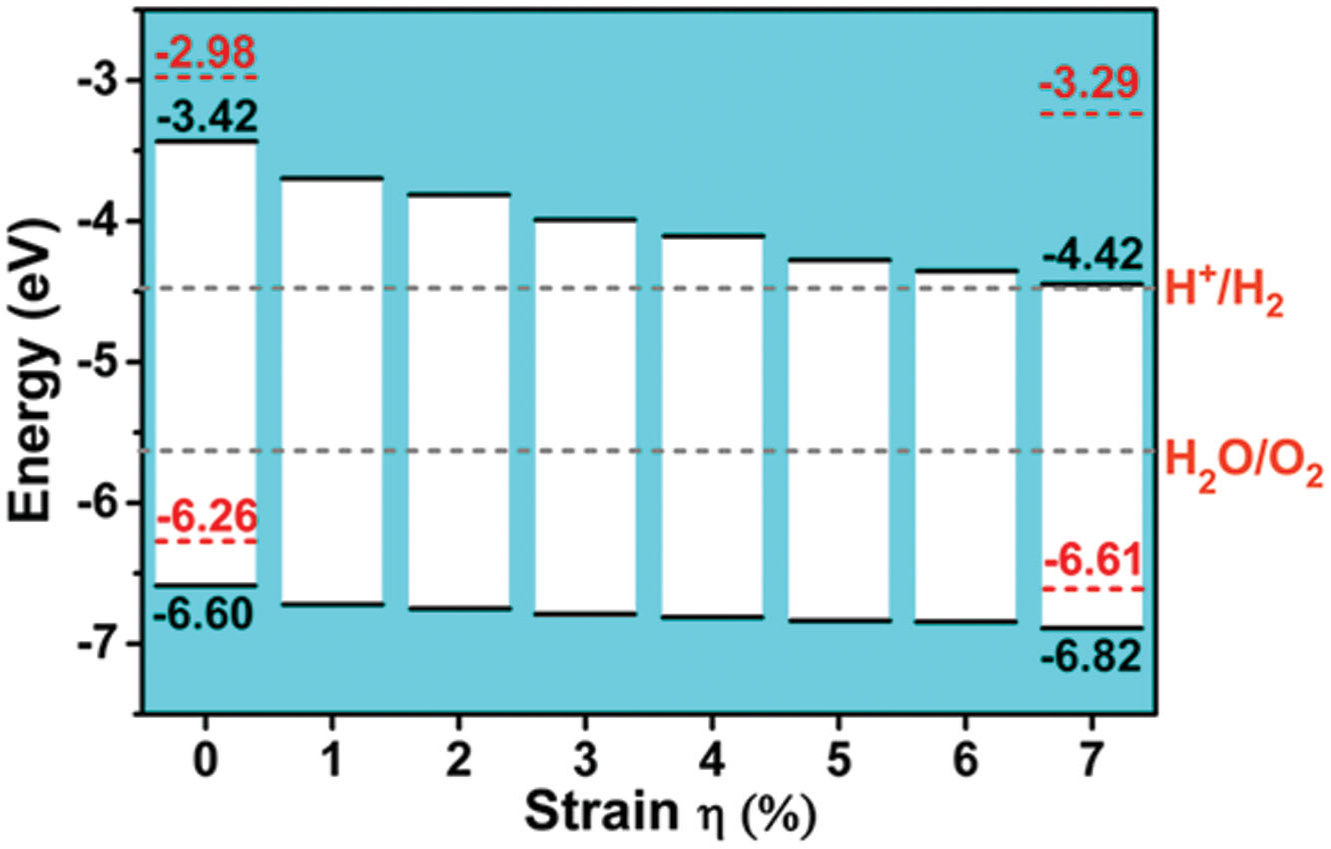
Calculated band alignment at the HSE06 level for the ph‐ZnSe (red dashed lines) and the t‐ZnSe (black lines) monolayers. The gray dashed lines represent the redox potential of water splitting reaction relative to the vacuum potential level.

To effectively photocatalyze the splitting of water, suitable alignment of the band edge to straddle the redox potentials of water splitting reaction is an essential requirement.^39^, ^40^ The standard water redox potential is used for discussion as done in previous studies.^41^, ^42^, ^43^ Here, we show that the HSE06‐based band alignment of the t‐ZnSe monolayer under various biaxial strains from 0% to 7% meets the requirement for photocatalytic water splitting. Note that compared to the PBE functional used by Tong et al., the HSE06 functional is expected to be more accurate as discussed above. As shown in **Figure** **3**, for the strain‐free t‐ZnSe monolayer, the energy level of VBM is at −3.42 eV, which is ≈1.02 eV higher than the water reduction potential (H^+^/H_2_ potential). With increasing the strain, the VBM level shifts downwards and approaches to the H^+^/H_2_ potential. However, for the t‐ZnSe monolayer under the strain up to 6% and 7%, the VBM energy level is still located about 0.10 and 0.02 eV above the H^+^/H_2_ potential, respectively. Meanwhile, the downward shift of the energy level of the conduction band minimum (CBM) induced by the strain ensures the alignment of the CBM below the water oxidation potential (H_2_O/O_2_ potential). So for the t‐ZnSe monolayer at the strain from 0% to 7%, alignment of the band edge ensures the redox potential of the water splitting located inside the bandgap. Such a desirable band edge alignment renders the t‐ZnSe monolayer a possible effective photocatalyst, e.g., not only as an ultraviolet‐light‐driven but also visible‐light‐driven photocatalyst for water splitting reaction.

### 2.3 Effective Mass of Carriers

**Figure 4:**
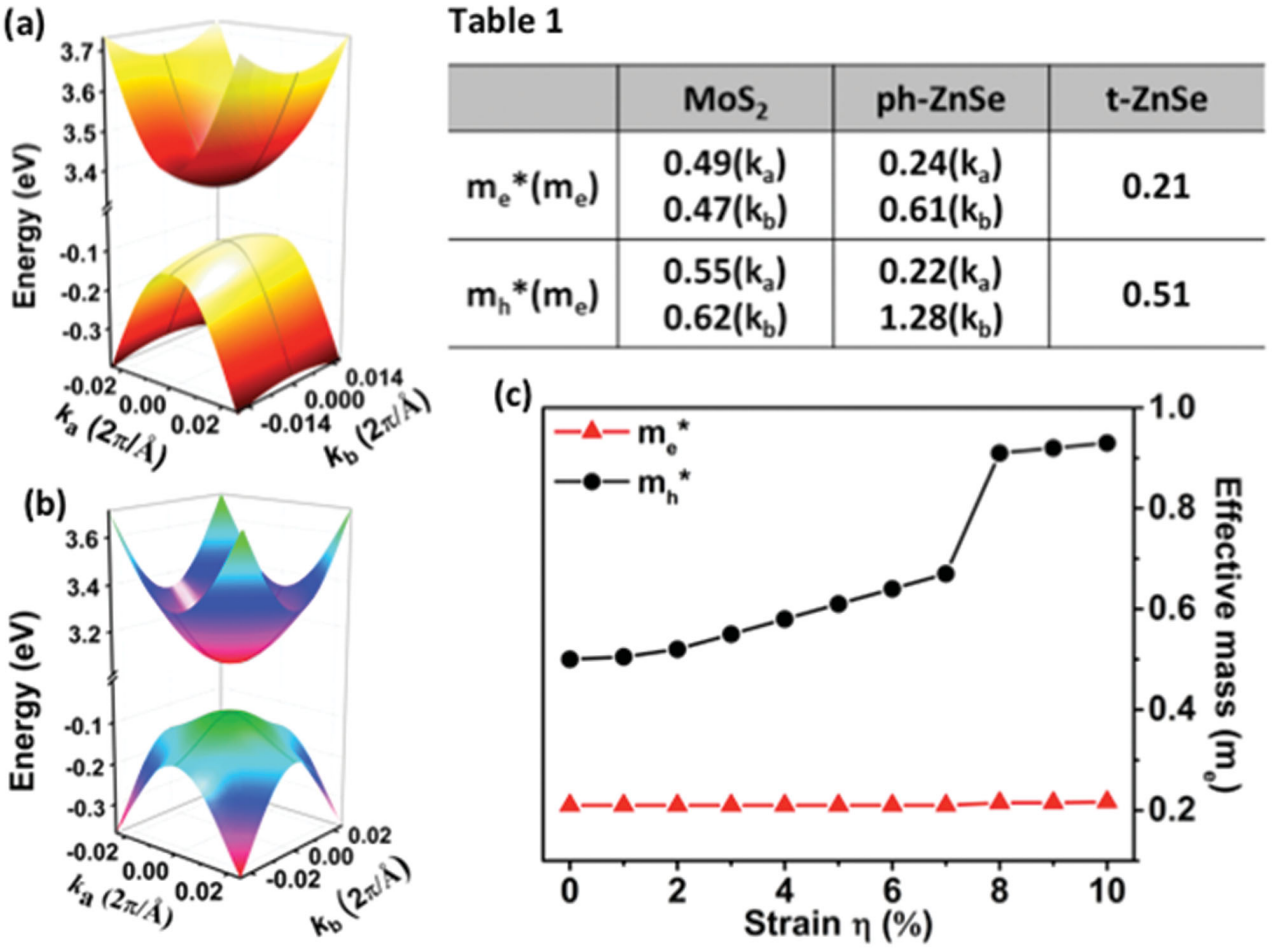
a,b) Surface plot of valleys around VBM and CBM for the ph‐ZnSe and the t‐ZnSe monolayers, respectively. Computed effective mass of electrons and holes for MoS_2_, ph‐ZnSe and t‐ZnSe monolayers are given in Table 1, where *k_a_* and *k_b_* refer to directions Γ → X and Γ → Y, respectively. c) Computed effective mass of electron (red triangle) and hole (black solid circle) versus the biaxial strain.

We also compute the effective mass of electron and hole in both ph‐ and t‐ZnSe monolayers. For the purpose of comparison, the effective mass of electron and hole in the MoS_2_ monolayer is also computed. As shown in Table 1 of **Figure** **4**, the carriers in the MoS_2_ monolayer entail an effective mass of ≈0.49/0.47 (*k_a_*/*k_b_*) and 0.55/0.62 (*k_a_*/*k_b_*) *m*_e_ for electron and hole, respectively, consistent with previous study.^44^ For the ph‐ZnSe and t‐ZnSe monolayers, plots of the 3D valley around the VBM and CBM are shown in Figure 4. Near the Γ point, drift of electrons and holes along *k_a_* (Γ → Χ) and *k_b_* (Γ → Y) is highlighted as gray lines. For the ph‐ZnSe monolayer, both electrons and holes have much smaller effective mass in *k_a_* direction with values of 0.24 and 0.22 *m*_e_, respectively. The relatively small carrier effective mass is consistent with the high carrier mobility reported by Sun et al. On the other hand, carriers with relatively large effective mass (*m*_e_ * = 0.61 *m*_e_ and *m*_h_ * = 1.28 *m*_e_) may reduce the drift of carriers in *k_b_* direction. Hence, fabrication of high‐performance electronic or optical devices based on the ph‐ZnSe monolayer may require control of current direction due to the anisotropic effective mass of carriers.

Contrary to the ph‐ZnSe monolayer, the t‐ZnSe monolayer exhibits isotropic effective mass in both *k_a_* and *k_b_* directions. As shown in Table 1, the effective mass of electron (0.21 *m*_e_) is less than half of that of MoS_2_ monolayer, and is comparable to that of electron in ph‐ZnSe monolayer. The computed electron mobility of t‐ZnSe is ≈138.8 cm^2^ V^−1^ s^−1^, about twice as large as that of MoS_2_ (see Table S2, Supporting Information). More remarkably, the strain has little effect on the effective mass of electron (*m*_e_ * = 0.22 *m*_e_ even at strain *η* = 10%), suggesting that the t‐ZnSe monolayer can serve as an n‐type channel material in FETs. The effective mass of hole is similar to that in the MoS_2_ monolayer (*m*_h_ * = 0.51 *m*_e_) so that the t‐ZnSe monolayer has a lower hole mobility than that of MoS_2_ due to the electron‐acoustic phonon coupling (Table S2, Supporting Information). In particular, because of the appearance of VBM around the K point, the effective mass of hole jumps to ≈0.92 *m*_e_ if the strain exceeds 7%, which leads to further decrease of the hole mobility. However, the electron still retains its effective mass of ≈0.22 *m*_e_, resulting in a high mass‐ratio of *m*_h_*/*m*_e_* ≈4.22. The high mass‐ratio of *m*_h_*/*m*_e_* implies potential application of the t‐ZnSe monolayer in switch devices or tunable *n*‐type devices.

### 2.4 Structure Transition from Pseudohexagonal to Tetragonal ZnSe: Born–Oppenheimer Molecular Dynamics Simulations

We have shown superiority of the t‐ZnSe monolayer over the previously synthesized ph‐ZnSe monolayer due to its strain‐dependent electronic and optical properties. An open question is whether the t‐ZnSe monolayer can be fabricated in the laboratory. Here, we perform BOMD simulations to show a possible fabrication route to produce t‐ZnSe monolayer from the pre‐synthesized ph‐ZnSe monolayer in the laboratory. In the BOMD simulations, we select a (39.65 × 40.92 Å^2^) supercell of the ph‐ZnSe monolayer as the initial configuration. Next, five different lateral pressures (100, 300, 500, 700, and 900 MPa) are chosen with temperature controlled at 300 K for the five independent simulations. As shown in the snapshots (see Figure S5, Supporting Information), at relatively low pressures (100 and 300 MPa) the pseudohexagonal structure is largely unchanged despite of slight expansion of the supercell along the *b* direction. The positions of the first and the second peak in the pair distribution function (*g*(*r*)) of the Zn–Se distance indicate that the nearest and next nearest Zn–Se distance entails the value ≈2.413 and 4.646 Å (see the upper panel in Figure S6, Supporting Information), respectively, consistent with previous experimental data.^29^

**Figure 5:**
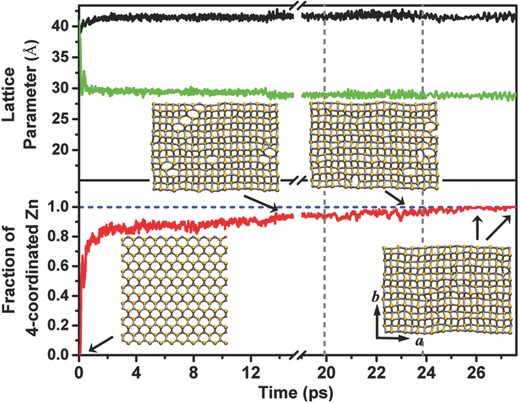
The black and the green lines present the evolution of the lattice parameters “*a*” and “*b*” of the 10 × 7 supercell, respectively, during the BOMD simulation with the lateral pressure fixed at 700 MPa. The lower panel illustrates the variation of the fraction of the four coordinated Zn atoms out of the total number of Zn atoms in the supercell. From left to right, the three time intervals separated by the two vertical gray dashed lines correspond to three sequential BOMD simulations at a fixed pressure (700 MPa) with temperature controlled at 300, 800, and 600 K, respectively. Insets are four snapshots of the lattice structures at different time stages of the three sequential simulations.

Interestingly, increasing the lateral pressure to 500 MPa leads to the transition of the ZnSe monolayer from the pseudohexagonal to the tetragonal structure. As shown in Figure S7a (Supporting Information), the lattice parameter along the *b* direction is reduced by ≈20%, concomitant with the rising of the tetragonal structure (Figure S7b, Supporting Information). Further increase of the lateral pressure to 700 MPa induces the rapid shrinkage of the ZnSe monolayer along the *b* direction by ≈30% (the upper panel in **Figure** **5**) while little change is seen in the *a*‐direction. The drastic reduction of the ZnSe monolayer along the *b* direction leads to the formation of extra Zn–Se bonds, a manifestation of the formation of tetracoordinated Zn atoms (the top‐left inset image in Figure 5). Time variation of the ratio of the number of the four‐coordinated Zn atoms over the total number of the Zn atoms is depicted in the lower panel of Figure 5. In the initial pseudohexagonal structure, all Zn atoms are surrounded by three nearest Se atoms. Within 1 ps, ≈80% of Zn atoms become tetracoordinated due to the formation of the extra Zn–Se bonds. At ≈2 ps, ≈90% of total Zn atoms become tetracoordinated. Beyond 2 ps, the ZnSe monolayer maintains the tetragonal structure but with a few hexagonal defects. Lastly, as in the case of 700 MPa pressure, the ZnSe monolayer under 900 MPa lateral pressure also undergoes a fast transition from pseudohexagonal to tetragonal structure (Figure S7a,c, Supporting Information). Dynamics of the structure transition can be viewed in Movies S3 and S4 (Supporting Information), taken from the 700 and 900 MPa simulations, respectively.

Our simulations show that at relatively high pressures (≥700 MPa), the pseudohexagonal structure can be easily transformed to the tetragonal structure. However, there still exist ≈10% hexagonal defects within the ZnSe monolayer after ≈20 ps simulation at 300 K and 700 MPa (see the top‐left inset images in Figure 5). Given the energetic favorability of the t‐ZnSe monolayer over ph‐ZnSe monolayer, such hexagonal defects are expected to be removed after long simulation. To speed up the transformation process, we undertake a simulated annealing experiment by taking the defect‐containing t‐ZnSe monolayer obtained at 300 K as the initial structure. We then run two sequential simulations with temperature controlled at 800 K for 3.90 ps and then at 600 K for 2 ps (corresponding to the last two time intervals in Figure 5). At 800 K, about a half number of hexagonal defects are removed (Movie S5, Supporting Information), while at the 600 K, the remaining hexagonal defects are removed (Movie S6, Supporting Information). Note that in realistic experiments, the compressive lateral pressure can be effectively applied to the 2D layered materials via using the hydrostatic pressure approach as reported by Dou et al. and Nayak et al.^45^, ^46^

The structure transition observed in our simulations suggests a possible fabrication route for making the t‐ZnSe monolayer from the ph‐ZnSe monolayer. Moreover, the superior properties of the t‐ZnSe monolayer also demand experimental feasibility of application of the large strain (up to 10%). To demonstrate such feasibility, we compute the tensile‐stress‐strain curve (see Figure S8, Supporting Information) and in‐plane stiffness (Table S2, Supporting Information) of the t‐ZnSe monolayer, and compare these results with those of the MoS_2_ monolayer. As shown in Figure S8 (Supporting Information), the continuous increase of the tensile stress with the strain up to 16% suggests that the t‐ZnSe monolayer can be stretched by 10% or larger without inducing a phase change. In addition, the computed in‐plane stiffness (44.17 N m^−1^) of the t‐ZnSe monolayer is much smaller than those of the MoS_2_ monolayer (131.97 and 129.28 N m^−1^ along zigzag and armchair direction, respectively), suggesting that the t‐ZnSe monolayer is mechanically much less rigid than the MoS_2_ monolayer. Note that the latter can be strained by as large as ≈11% in experiments.^18^, ^47^ So we expect that the much less rigid t‐ZnSe monolayer could be strained by up to 10% as well, using the same experimental approach as for the MoS_2_ monolayer.^18^, ^47^

## 3 Conclusion

We have shown that the ZnSe monolayer with tetragonal structure is the most stable structure based on a global minimum search. Three theoretical results indicate likelihood of future experimental synthesis of the t‐ZnSe monolayer: (1) lower energy than the experimentally prepared ph‐ZnSe monolayer, (2) dynamic and thermal stability demonstrated by phonon‐spectrum computation and BOMD simulation, and (3) simulation evidence of pressure‐induced structure transition from pseudohexagonal to tetragonal structure. In view of the fact that the ph‐ZnSe has been successfully fabricated in the laboratory, experimental realization of the t‐ZnSe monolayer appears to be feasible via the application of hydrostatic pressure onto the presynthesized ph‐ZnSe monolayer.^29^

A good agreement between the computed bandgap (≈3.40 eV) with the measured one (≈3.50 eV) lends credibility of the selected DFT method for computing electronic properties of the t‐ZnSe polymorph. Our DFT computation predicts that the bandgap of t‐ZnSe monolayer is sensitive to the external biaxial strain but can maintain its direct semiconducting properties under strain up to 7%. Further increase of the strain can reduce the bandgap to red‐light region. The ph‐ZnSe monolayer, however, tends to retain its bandgap value of ≈3.30 eV even at strain up to 10%. The highly tunable bandgap from blue‐light region to red‐light region suggests potential application of the t‐ZnSe monolayer as an LED material. The computed optical absorption spectrum also confirms that optical absorption of the t‐ZnSe monolayer can be shifted into visible‐light region via applying strain. Moreover, the band edge of strained t‐ZnSe monolayer can well straddle the redox potential of the water splitting reaction. As such, strained t‐ZnSe monolayer may be utilized as a visible‐light photocatalyst for splitting water. Finally, the predicted optical absorption in the blue–yellow region suggests potential application of the t‐ZnSe monolayer in multijunction solar cells for enhancing the efficiency of photoelectric conversion.

## 4 Experimental Section

*Global Minimum Search*: In our search for the global minimum structure of 2D ZnSe monolayer, population size of each generation was set to be 30, and the number of generations was fixed to be 30. In the ensuing generations, 60% of the population was generated from the best (lowest‐energy) structures in the previous generation by using the particle‐swarm optimization scheme and the other 40% was generated randomly to ensure diversity of the population. Local optimization including the atomic positions and lateral lattice parameters was performed for each of the initial structures. The generalized gradient approximation (GGA) in the PBE form was used for the structure relaxation and total‐energy computation.^48^, ^49^ A dense *k*‐point sampling with the grid spacing less than 2π × 0.04 Å^−1^ in the Brillouin zone was selected. An 18 Å vacuum spacing was set along the *z*‐direction so that interaction between the adjacent solid sheets could be neglected. The unit cells with 2, 4, and 8 atoms were considered.

*DFT Computation**:*** The plane‐wave basis sets and projected augmented wave (PAW) pseudopotentials implemented in the Vienna ab initio simulation package (VASP 5.3) were used to perform geometry optimization and calculation of electronic and optical properties using DFT methods.^50^, ^51^, ^52^, ^53^ The GGA with Perdew and Wang's 1991 (PW91) functional was selected to treat the exchange‐correlation interaction.^54^, ^55^, ^56^, ^57^ The energy cutoff for the plane‐wave basis sets was set as 300 eV which was tested large enough for the total energy calculation and the prediction of the electronic structure of the systems (Figure S9, Supporting Information). Spin‐restricted calculations were adopted for ZnSe monolayer with 10 × 10 × 1 Monkhorst–Pack meshes, respectively. All the structures were fully relaxed until the force on each atom was smaller than 0.01 eV Å^−1^ at the PW91 level. To study the strain effect on ZnSe monolayer, a given equivalent biaxial strain *η* was imposed to the in‐plane lattice parameter along both *a* and *b* directions. The electronic properties of ZnSe monolayer were computed using the HSE06 functional with consideration of the spin‐orbit coupling (SOC) effect.^58^ The inclusion of the SOC effect resulted in a decreased bandgap by ≈0.16 eV and the splitting of the valence band at the Γ point (see Figure S10a, Supporting Information). The latter was important to the prediction of electronic properties and the effective mass of carriers. Based on the HSE06 functional with the SOC effect, the computed bandgap of the bulk ZnSe was 2.25 eV, consistent with the value based on the GW computation, reported by Zhou et al.^31^ For 2D materials, the bandgap computed at the HSE06 level was usually smaller than that computed at the GW level. Such discrepancy between the HSE06 and the GW methods was thoroughly discussed in previous studies. The HSE06/SOC bandgap was considered quite reasonable in view of good agreement with the photoluminescence energy.^8^, ^59^, ^60^

The absorption spectra were computed based on the frequency‐dependent dielectric function by using an expression reported in Qiao et al.^4^ The imaginary part of the dielectric function was derived by a summation over empty states; and the real part of the dielectric function was calculated via the Kramers–Kronig transformation. More detailed explanation can be found in Gajdos et al.^38^ As shown in Figure S10b (Supporting Information), the computed optical absorbance spectrum of the t‐ZnSe monolayer exhibits four peaks at 3.60, 3.87, 4.38, and 4.95 eV, respectively, consistent with those reported by Zhou et al.^31^ So as far as the trend of the bandgap and the shift of the absorbance peak as a function of the applied strain is concerned, the HSE06 method appears to be reasonable.

The carrier mobility (*μ*) of both t‐ZnSe and MoS_2_ was calculated on the basis of the deformation potential (DP) theory as proposed by Bardeen and Shockley,^61^ which had been widely used to estimate *μ* in the 2D materials.^62^, ^63^ Based on the DP theory, the carrier mobility can be computed from Equation (1) below

(1) $$\mu_{2D}=\frac{2e\hbar^{3}C^{2D}}{3k_{B}T{\left|m^{*}\right|}^{2}E_{1}^{2}}$$

where *m* ^*^, *T*, and *C* ^2D^ represent the effective mass of carrier, ambient temperature (*T* = 300 K), and in‐plane stiffness, respectively. The DP constant *E*_1_ is denoted as the strain‐induced shift of the band edges.

*BOMD Simulation*: BOMD simulation was performed on the basis of DFT method and in the form of PBE functional as implemented in the CP2K code.^37^ To treat the interaction between the valence electrons and atomic cores, a mixed Gaussian and plane‐wave basis set with the Goedecker–Teter–Hutter pseudopotential was adapted.^64^, ^65^ To test thermal stability of the t‐ZnSe monolayer, the *NVT* ensemble was chosen, and an 8 × 8 supercell with size 32.82 × 32.82 Å^2^ was used. For the study of the structure transition of the ZnSe monolayer, a 10 × 7 supercell of ph‐ZnSe monolayer with size 39.65 × 40.92 Å^2^ was applied and the *NPT* ensemble with temperature controlled at 300 K was selected. The time step was set as 1.0 and 0.5 fs for *NVT* and *NPT* simulations, respectively. In addition, the vacuum distance was set as 20 Å so that the interaction between neighboring monolayers could be neglected. Six trajectories of BOMD simulations are illustrated in Movies S1–S6 (Supporting Information), where the gray and orange spheres represent Zn and Se atoms, respectively.

## Supporting information

As a service to our authors and readers, this journal provides supporting information supplied by the authors. Such materials are peer reviewed and may be re‐organized for online delivery, but are not copy‐edited or typeset. Technical support issues arising from supporting information (other than missing files) should be addressed to the authors.

Supplementary

Supplementary

Supplementary

Supplementary

Supplementary

Supplementary

Supplementary
