# Supplementary material for: Simulation Evidence of Hexagonal‐to‐Tetragonal ZnSe Structure Transition: A Monolayer Material with a Wide‐Range Tunable Direct Bandgap
Source: Adv Sci (Weinh). 2015 Oct 28;2(12):1500290. doi: 10.1002/advs.201500290 (PMC5061093; doi:10.1002/advs.201500290)
Supplement: Supplementary file 1 — Supplementary [file ADVS-2-0l-s001.pdf]

Copyright WILEY-VCH Verlag GmbH & Co. KGaA, 69469 Weinheim, Germany, 2015.

ADVANCED SCIENCE
Open Access

Supporting Information

for *Adv. Sci.*, DOI: 10.1002/advs.201500290

Simulation Evidence of Hexagonal-to-Tetragonal ZnSe Structure Transition: A Monolayer Material with a Wide-Range Tunable Direct Bandgap

*Lei Li, Pengfei Li, Ning Lu, Jun Dai, and Xiao Cheng Zeng**

Supporting Information

Simulation evidence of hexagonal-to-tetragonal ZnSe structure transition: a monolayer material with wide-range tunable direct bandgap

Lei Li[†], Pengfei Li[⊥,†], Ning Lu[†], Jun Dai[†], Xiao Cheng Zeng[†,⊥]*

[†]Department of Chemistry, University of Nebraska-Lincoln, Lincoln, Nebraska 68588, United States

[⊥]Department of Chemical Physics, Hefei National Laboratory for Physical Sciences at Microscale and Collaborative Innovation Center of Chemistry for Energy Materials, University of Science and Technology of China, Hefei, Anhui 230026, China

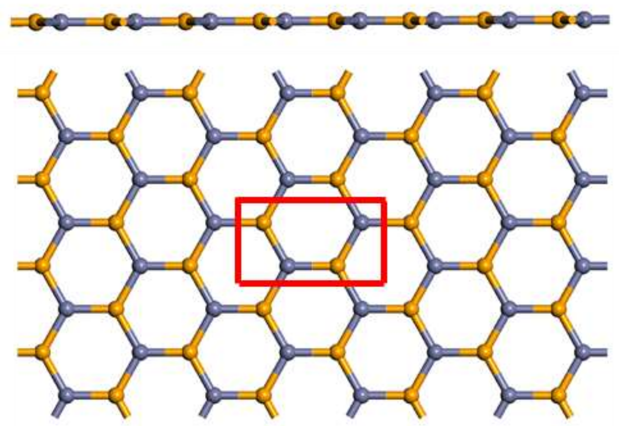

Figure S1. Side and top views of the planar structure (the third lowest structure) of the hexagonal ZnSe monolayer. The unit cell is highlighted in red rectangle. Zn and Se atoms are represented with orange and gray spheres.

Table S1. Lattice parameters of the optimized ph-ZnSe and t-ZnSe monolayers at PW91 level. Definition of the lattice parameter *a* and *b* is given in Figure 1. The total energies relative to the ph-ZnSe monolayer computed at the PW91 and HSE06 levels are given in the third and the fourth column, respectively.

	Lattice Parameter/Å	PW91/meV	HSE06/meV
ph-ZnSe	a=3.965<br>b=5.846	0.0	0.0
t-ZnSe	a=b=4.103	-28.0	-54.0

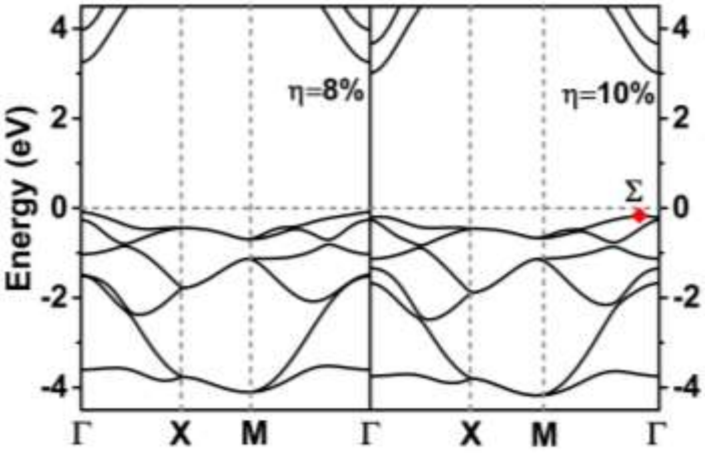

Figure S2. Computed band structures of the ph-ZnSe monolayer at 8% (left panel) and 10% (right panel) biaxial strain. The symbol 'Σ' refers to the point (1/5, 1/5, 0.0).

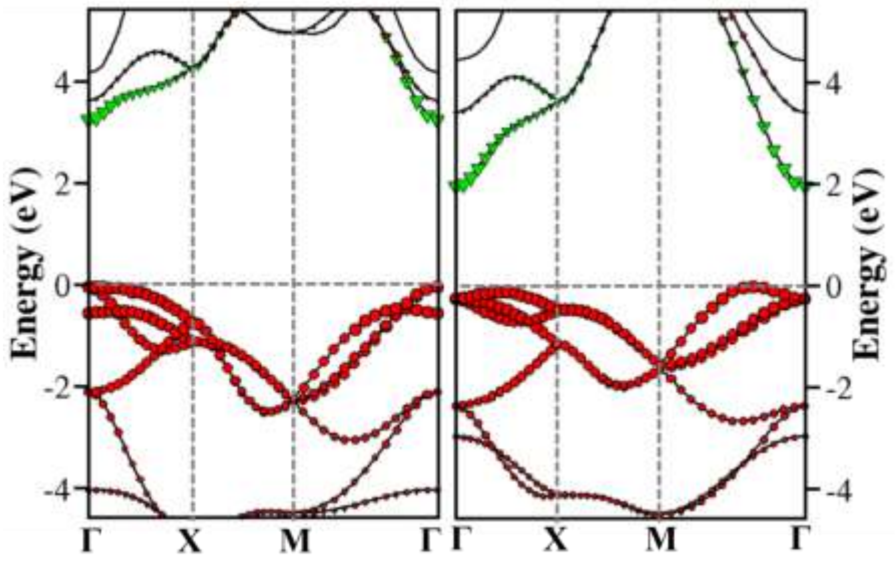

Figure S3. Computed HSE06 band structures of the stain-free (left panel) and 10%-strained (right panel) t-ZnSe monolayer. The green triangle and the red circle represent the contribution from the Zn *s* states and the Se *p* states, respectively.

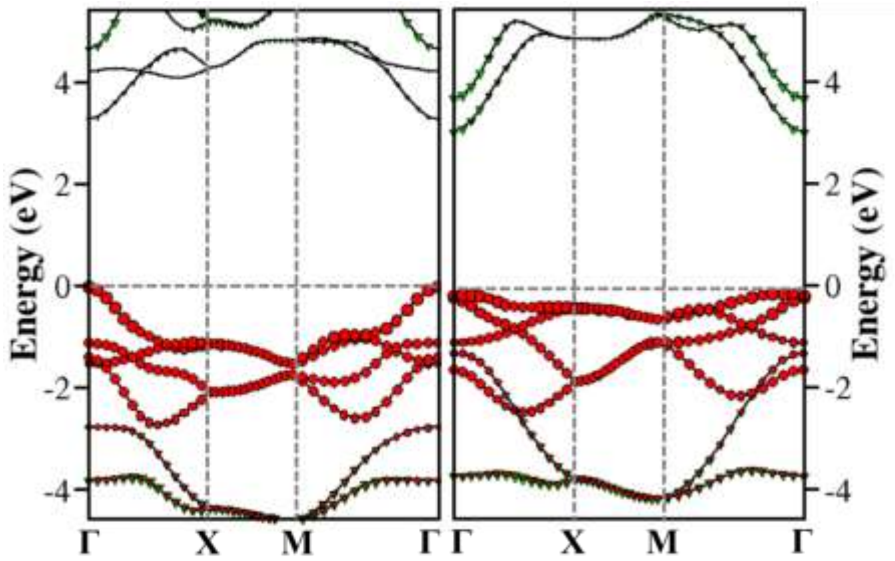

Figure S4. Computed HSE06 band structures of the stain-free (left panel) and 10%-strained (right panel) ph-ZnSe monolayer. The green triangle and the red circle represent the contribution from the Zn *s* states and the Se *p* states, respectively.

Table S2. Deformation potential E_1, in-plane stiffness C^{2D}, effective mass $m*$ and mobility for electron (e) and hole (h) along k_a direction (armchair growth direction) in the MoS_2 monolayer and k_a or k_b ($k_{a/b}$) direction in the t-ZnSe monolayer at 300 K. C^{2D} along zigzag growth direction for MoS_2 is 131.97 N/m.

	Carrier type	E_1 (eV)	C^{2D}(N/m)	$m*$(m_e)	μ (cm^2 V^{-1} s^{-1})
MoS_2	e(k_a)	-10.85	129.28	0.49	74.25
	h(k_a)	-5.49	129.28	0.55	202.85
t-ZnSe	e($k_{a/b}$)	-10.16	44.17	0.21	138.81
	h($k_{a/b}$)	-4.90	44.17	0.51	101.18

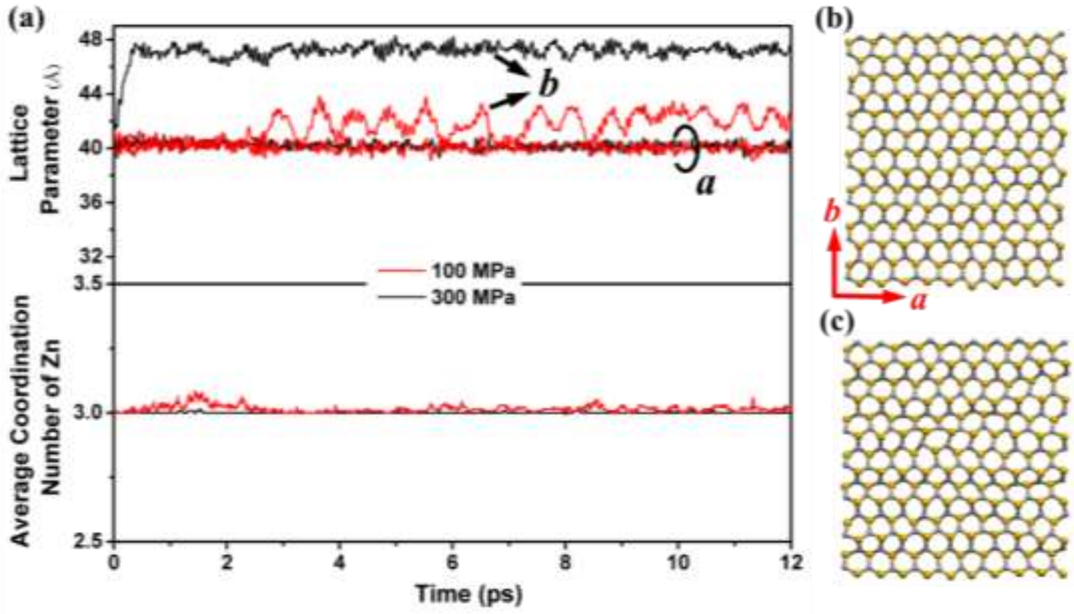

Figure S5. (a) The upper panel presents time evolution of the lattice parameter of the 10 × 7 supercell during the BOMD simulation. "*a*" and "*b*" denote the lattice parameter directions (in ph-ZnSe monolayer, "*a*" and "*b*" correspond to zigzag and armchair growth directions, respectively). The lower panel illustrates variation of the coordination number of the Zn atoms as a function of time. The snapshots at 12 ps from the BOMD simulations (with pressure controlled at 100 and 300 MPa, respectively) are displayed in (b) and (c).

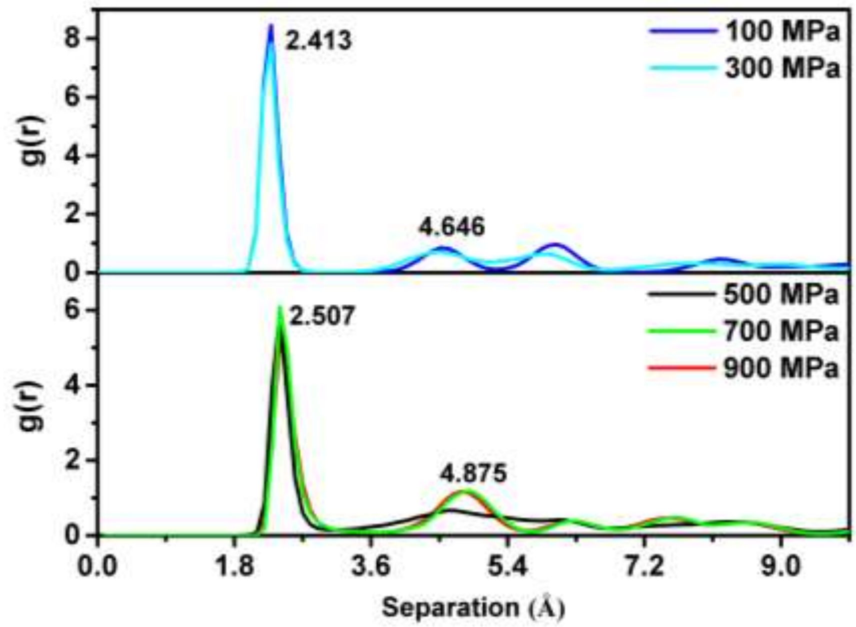

Figure S6. Pair distribution function (g(r)) of the Zn-Se distance from the BOMD simulations with pressure controlled at 100, 300, 500, 700 and 900 MPa, respectively. The inset numbers indicate the positions of the first or the second peaks.

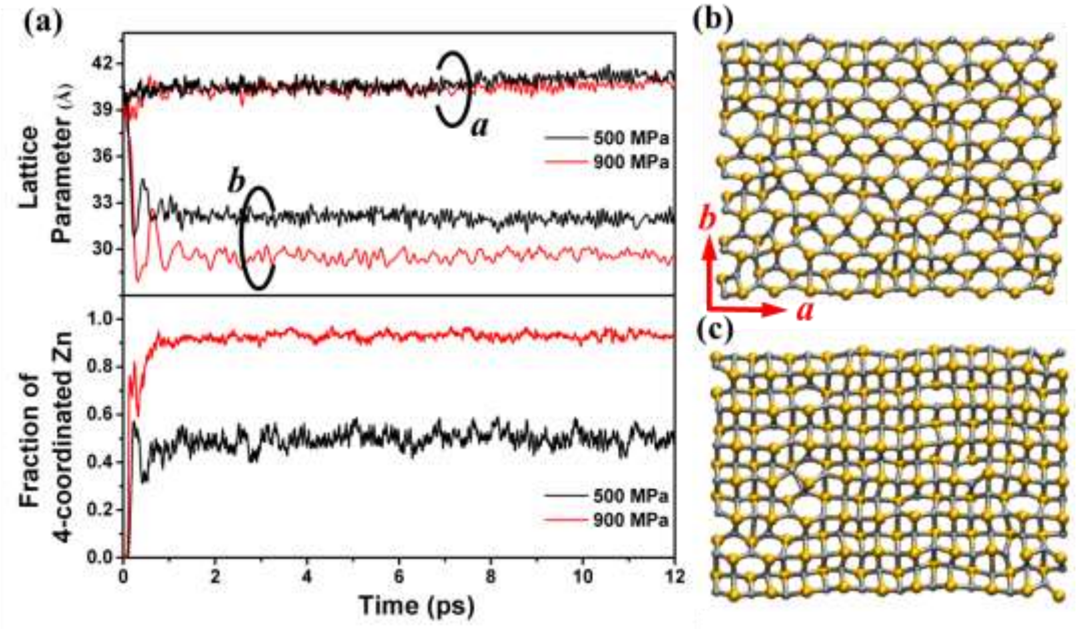

Figure S7. The upper panel presents the evolution of the lattice parameter of the 10 × 7 supercell during the BOMD simulation. "*a*" and "*b*" indicate the lattice parameter directions (in ph-ZnSe monolayer, "*a*" and "*b*" correspond to zigzag and armchair growth direction, respectively). The lower panel illustrates the variation of the fraction of the four-coordinated Zn atoms out of the total number of Zn atoms in the supercell. The snapshot structures at 12 ps from BOMD simulation with the lateral pressure fixed at 500 and 900 MPa are displayed in (b) and (c), respectively.

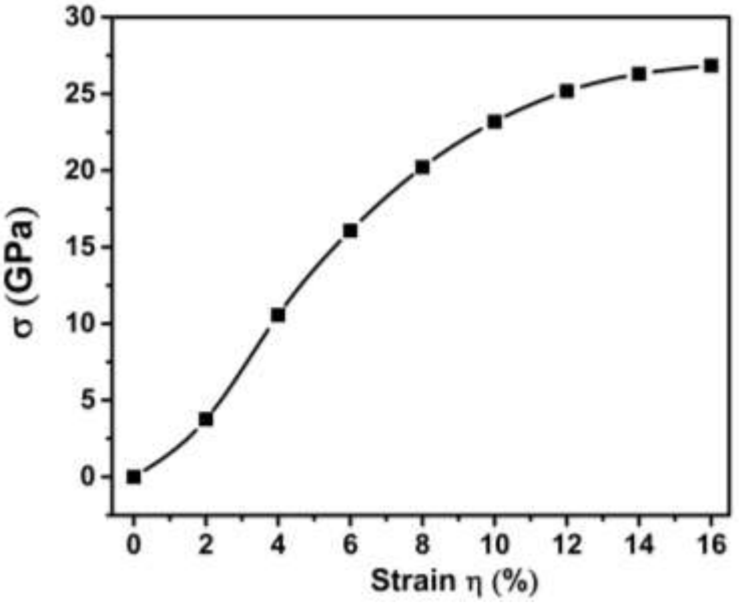

Figure S8. Variation of tensile stress with the strain applied along *a* (or *b*) direction of the t-ZnSe monolayer.

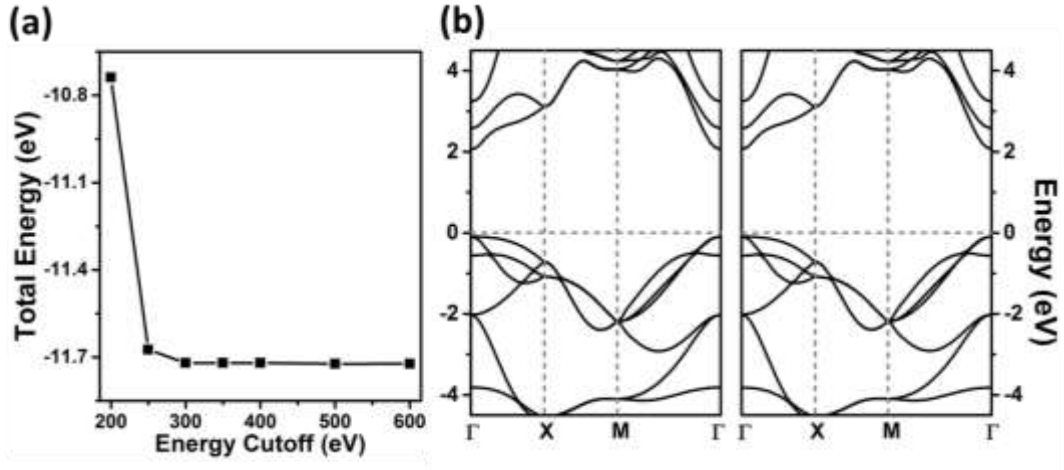

Figure S9, (a) Computed energy of the t-ZnSe unit cell versus the kinetic energy cutoff. (b) Computed band structure of the t-ZnSe monolayer at the PW91 level with kinetic energy cutoff set as 300 eV (left panel) and 600 eV (right panel), respectively.

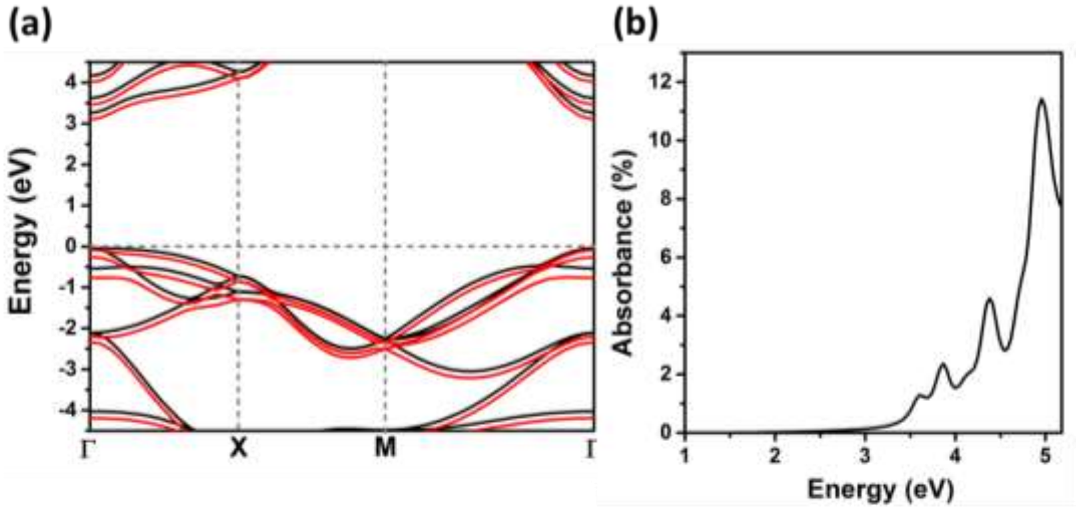

Figure S10. (a) Computed HSE06 band structures of the t-ZnSe monolayer with (red line) and without (black line) considering the SOC effect. (b) Computed optical absorbance spectra for the strain-free t-ZnSe monolayer using HSE06_RPA method.
